# AAAS joins the Translational Medicine family

**DOI:** 10.1186/1479-5876-7-32

**Published:** 2009-05-07

**Authors:** Christian Brander, Francesco M Marincola

**Affiliations:** 1 Laboratori de Retrovirologia, Fundació irsiCaixa Hospital Universitari Germans Trias i Pujol Ctra del Canyet s/n 08916 Badalona, and Institució Catalana de Recerca i Estudis Avançats, Barcelona, Catalonia, Spain; 2 Infectious Disease and Immunogenetics Section (IDIS), Department of Transfusion Medicine, Clinical Center, and Center for Human Immunology (CHI), National Institutes of Health, Bethesda, Maryland, 20892, USA

## Abstract

The AAAS has announced the launch of *Science Translational Medicine*. This is further and critical recognition of this discipline and we are deeply gratified that translational medicine has risen to the level of recognition by one of the world's most prestigious scientific organizations. We believe that *Science Translational Medicine *will provide another valuable venue for the rapid and broad dissemination of important articles in the field and contribute to enhancing the effectiveness of translational medicine overall.

It has been almost six years since we launched the *Journal of Translational Medicine *as an open-access journal with Biomed Central [1]. At the beginning, we faced the inevitable skepticism and received several inquires among others also from *Science *reporters questioning both the significance of translational medicine in today's biomedical world and the need for a new journal dedicated to it.

## Editorial

Some suggested that translational medicine (aka translational research/translational science) was a fad: a term fabricated to divert support from the basic sciences on the one hand and/or from clinical research on the other and, even more critical, a synonym for scientific investigations of lower quality that tried to excuse the supposed lack of scientific rigor with inherit disadvantages and limitations often not encountered for instance in studies using small laboratory animals.

We however argued that although bridging the gap between discovery and clinical application was not an intrinsically novel concept – it does after all represent the long term goal of most scientists and clinicians – the value in identifying it as a distinct field was largely in enhancing the effectiveness of methods to efficiently transfer knowledge from the bench to the bedside, and, importantly, from the bedside to the bench [2-4]. In particular, we suggested that "the traditional goals of biomedical research function as a substrate for the catalytic activity of translational research that, like an enzyme, is aimed at enhancing the efficiency rather than modifying the process" [3].

We also argued, in response to editors and publishers of existing journals who questioned the need for yet another one, that *JTM *would uniquely advance translational medicine through:

**1) Open access **– Thanks to the partnership and vision of BioMed Central, information is divulged rapidly, broadly and without barriers to create an interactive platform for scientists, clinicians, patients, funding agencies and regulatory agencies [5].

**2) A specialized Editorial Board **consisting exclusively of translational scientists and physicians with expertise in both the clinic and in the laboratory. We felt that only individuals with combined expertise could authoritatively and constructively discriminate between good and bad clinical science. Clinical studies are often limited by necessity due to practical, financial and ethical constraints; yet they have tremendous value because they provide insights to the actual reality of disease [1]. These insights can often times not be gained in in-vitro analyses only or by experimentation in animal models. Thus, the Board was selected not only according to individual achievement but also according to their demonstrated enthusiasm and dedication to the field, knowing that many board members would face some sort of skepticisms by their peers.

**3) An equal interest in descriptive and mechanistic studies**. It is our conviction that knowledge about human disease is limited and a bottom-up inductive approach (i.e. assessing human diseases in patients and patient samples) represents a preliminary step that should be valued as much as elegant mechanistic studies, which can be of limited relevance to human disease [6].

The *Journal of Translational Medicine *has done so well in part because the significance of this emerging discipline has become widely accepted. We contend that translational medicine will be increasingly central to the identification of novel products with potential therapeutic value. Translational strategies may respond to the need to test novel therapies with solutions that address the enormous costs of clinical research, the lack of reliable pre-clinical models that could help prioritize products for clinical testing, the lack of predictive biomarkers that could help patient selection and the lack of surrogate biomarkers that could help with the assessment of product efficacy early in development.

All of us who have been dedicated from the beginning to this important field applaud the recent announcement by the American Association for the Advancement of Science that it is launching *Science Translational Medicine *[7]. We are deeply gratified that translational medicine has risen to the level of recognition by one of the world's most prestigious scientific organizations. Yet, *Science Translational Medicine *will actually be a closed access title, quite a significant obstacle to the discipline-barrier-breaking spirit which progress in translational research depends on. We reiterate the benefits of openness – not least as evidenced by the NIH Open Access policy which is now both mandatory and non-time-limited – which JTM offers. Nevertheless, we believe that *Science Translational Medicine *will provide another valuable venue for the rapid and broad dissemination of important articles in the field and contribute to enhancing the effectiveness of translational medicine overall.
